# Application of Collagen-Model Triple-Helical Peptide-Amphiphiles for CD44-Targeted Drug Delivery Systems

**DOI:** 10.1155/2012/592602

**Published:** 2012-11-14

**Authors:** Margaret W. Ndinguri, Alexander Zheleznyak, Janelle L. Lauer, Carolyn J. Anderson, Gregg B. Fields

**Affiliations:** ^1^Department of Biochemistry, University of Texas Health Science Center, 7703 Floyd Curl Drive, San Antonio, TX 78229, USA; ^2^Departments of Chemistry and Biology, Torrey Pines Institute for Molecular Studies, 11350 SW Village Parkway, Port St. Lucie, FL 34987, USA; ^3^Mallinckrodt Institute of Radiology, Washington University School of Medicine, 510 S Kingshighway Boulevard, St. Louis, MO 63110, USA; ^4^Department of Molecular Therapeutics, Scripps Florida, 130 Scripps Way, Jupiter, FL 33458, USA; ^5^Department of Radiology, University of Pittsburgh, 200 Lothrop Street, Pittsburgh, PA 15219, USA

## Abstract

Cancer treatment by chemotherapy is typically accompanied by deleterious side effects, attributed to the toxic action of chemotherapeutics on proliferating cells from nontumor tissues. The cell surface proteoglycan CD44 has been recognized as a cancer stem cell marker. The present study has examined CD44 targeting as a way to selectively deliver therapeutic agents encapsulated inside colloidal delivery systems. CD44/chondroitin sulfate proteoglycan binds to a triple-helical sequence derived from type IV collagen, **α**1(IV)1263–1277. We have assembled a peptide-amphiphile (PA) in which **α**1(IV)1263–1277 was sandwiched between 4 repeats of Gly-Pro-4-hydroxyproline and conjugated to palmitic acid. The PA was incorporated into liposomes composed of DSPG, DSPC, cholesterol, and DSPE-PEG-2000 (1 : 4 : 5 : 0.5). Doxorubicin-(DOX-)loaded liposomes with and without 10% **α**1(IV)1263–1277 PA were found to exhibit similar stability profiles. Incubation of DOX-loaded targeted liposomes with metastatic melanoma M14#5 and M15#11 cells and BJ fibroblasts resulted in IC_50_ values of 9.8, 9.3, and >100 **μ**M, respectively. Nontargeted liposomes were considerably less efficacious for M14#5 cells. In the CD44^+^ B16F10 mouse melanoma model, CD44-targeted liposomes reduced the tumor size to 60% of that of the untreated control, whereas nontargeted liposomes were ineffective. These results suggest that PA targeted liposomes may represent a new class of nanotechnology-based drug delivery systems.

## 1. Introduction

The ultimate goal of targeted nanotechnology-based drug delivery systems (nanoDDSs) in cancer therapy is to improve the therapeutic index of cytotoxic agents by selectively increasing their concentration at the tumor site. Liposomes in particular have attracted much attention as site-specific drug delivery vehicles because of their biocompatibility [1, 2], and the ease with which they can be manipulated to accommodate targeting ligands to further increase the specificity and therefore the potency of encapsulated chemotherapeutics [3]. Numerous targeted liposomes have been developed and are in clinical trials [2].

The cell surface proteoglycan CD44 is overexpressed on a variety of tumor cells [4, 5], and cells with higher expression of CD44 have a greater migratory and invasive potential on hyaluronate-coated substrates [6]. In addition, 4- to 6-fold elevated CD44 expression is associated with tumor growth and metastasis [7]. CD44 interaction with hyaluronan induces ankyrin binding to MDR1 (P-glycoprotein), resulting in the efflux of chemotherapeutic agents and chemoresistance in tumor cells [8–10]. Interestingly, CD44 has been revealed as a cancer stem cell marker for numerous tumor types [5, 11–17]. A theory is emerging that CD44 positive cells within a tumor display true stem cell properties such that one cell can give rise to an entire tumor [12]. This makes the development of CD44-targeted drugs important as few therapeutics are capable of killing 100% of the cells within a tumor. 

Ligands that bind CD44 undergo endocytosis [18, 19], making this receptor a good candidate for targeted drug delivery [20–24]. CD44 in the chondroitin sulfate proteoglycan (CSPG) modified form is among the receptors uniquely overexpressed in metastatic melanoma [4]. Targeting strategies for drug delivery vehicles against the CD44 receptor in melanoma have included hyaluronan/hyaluronic acid (HA) and its fragments. HA liposomes containing DOX were previously shown to be significantly more effective than free DOX *in vitro* against B16F10 melanoma cells [21] and *in vivo* against a variety of mouse tumor models [22, 24]. HA liposomes have been used to effectively deliver mitomycin C *in vivo* in three mice tumor models [25] and antitelomerase siRNA *in vitro* to CD44-expressing lung cancer cells [26].

A possible disadvantage of using HA as a targeting ligand is that, as a high molecular weight species, it may be quickly removed from circulation by hepatic cells [27]. In an attempt to circumvent this possible problem, enzymatically degraded HA fragments of lower molecular weight (hexameric fragments) have been used by Eliaz and Szoka Jr. [20] as targeting moieties in DOX-loaded liposomes against the CD44-overexpressing B16F10 melanoma cells. The hexameric HA induced rapid dose-dependent CD44 receptor binding of the targeted liposomes to melanoma cells. However, the low molecular weight HA fragments were also found to have lower affinity to the CD44 receptor than the intact HA, thus diminishing the targeting capabilities. 

**Figure 1:**
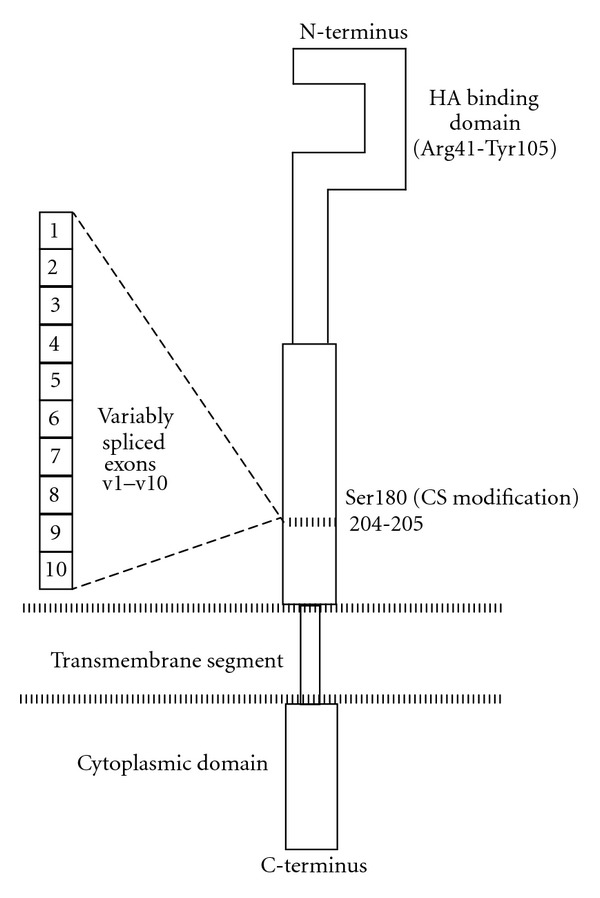
Schematic structure of CD44. The hyaluronate/hyaluronic acid (HA) binding site is in the *N*-terminal portion (Link module) of CD44 (residues Arg41-Tyr105) [33–35], while the CS modification primarily occurs at Ser180 [31]. The alternatively spliced variants of CD44 contain inserts at residues 204-205 of the parent protein [4]. Heparan sulfate modification occurs in exon v3 [36]; dermatin sulfate modification is observed for the nonspliced protein [37, 38], and CD176/Thomsen-Friedenreich antigen is found in spliced CD44 variants [39, 40]. The binding of *α*1(IV)1263–1277 to CD44 is dependent upon CS [41], and thus *α*1(IV)1263–1277 binding occurs in a region distinct from HA binding.

Unfortunately, an approach that employs HA and/or its fragments as the targeting moiety to CD44 suffers from reduced selectivity because other cell surface receptors such as RHAMM have been shown to bind HA just as avidly as CD44 [28, 29]. In addition, HA binding to CD44 is not sensitive to distinct glycosylation patterns of this receptor, as, for example, the site of chondroitin sulfate (CS) modification is distant from the HA binding site (Figure 1). HA modified delivery systems will bind to any cell that possesses CD44, as recently shown for macrophages [30]. Finally, CS modification of CD44 (which occurs in melanoma) negatively regulates HA binding [31, 32].

In addition to binding to HA, CS modified CD44 binds collagen [42–44]. The sequence to which CD44 binds within the type IV collagen triple helix has been identified as *α*1(IV)1263–1277 (gene-derived sequence Gly-Val-Lys-Gly-Asp-Lys-Gly-Asn-Pro-Gly-Trp-Pro-Gly-Ala-Pro) [41, 45]. Efficient binding is dependent upon CS modification of CD44 [41]. This sequence is not bound by collagen-binding integrins [41, 46]. We have previously constructed *α*1(IV)1263–1277 based triple-helical “peptide-amphiphiles” (PAs) [general structure C_n_-(Gly-Pro-Hyp)_4_-Gly-Val-Lys-Gly-Asp-Lys-Gly-Asn-Pro-Gly-Trp-Pro-Gly-Ala-Pro-(Gly-Pro-Hyp)_4_-NH_2_] specific for CD44/CSPG [41, 47–49]. M14#5 human melanoma cells bound to C_14_, C_16_, or C_18_ *α*1(IV)1263–1277 PA with EC_50_ approximately 0.08–0.5 *μ*M [41, 46, 50]. The amphiphilic design of the PA construct facilitates the anchoring of the functional “head group” of the construct to the liposome surface by the insertion of the hydrophobic acyl “tail” into the lipid bilayer. This in turn allows the hydrophilic head group or targeting the portion of the PA to protrude outward from the liposomal surface making it available to interact with the CD44/CSPG receptor. The incorporation of the *α*1(IV)1263–1277 PAs into rhodamine-loaded liposomes did not destabilize these systems and conferred targeting selectivity to liposomes against cell lines varying in the CD44 expression based on the receptor/PA ligand recognition [23].

**Scheme 1:**
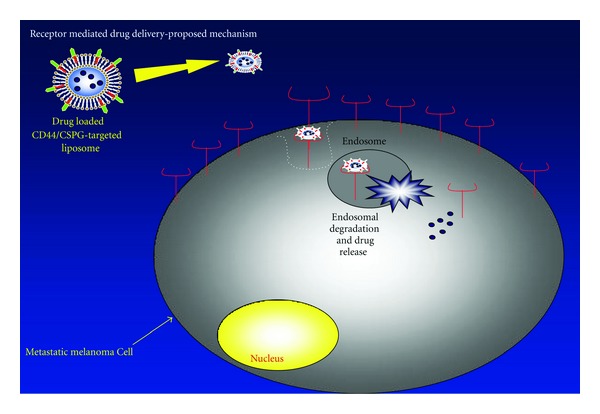
Schematic depiction of targeted liposomal delivery to CD44/CSPG metastatic melanoma cells. The *α*1(IV)1263–1277 PA (red alkyl tail and green peptide head group) is incorporated into liposomes along with DOX (blue circles). The liposome targets CD44/CSPG (red) on the melanoma cell surface. The liposome-receptor complex is internalized via endocytosis and DOX released. The mechanism of delivery was described previously [23]. This scheme does not explicitly propose how liposomes are trafficked through different intracellular compartments.

In the current study we evaluated the stability of distearoyl phosphatidylglycerol-(DSPG-)distearoyl phosphatidylcholine (DSPC) DOX-loaded liposomes both with and without the *α*1(IV)1263–1277 PA. We incorporated PEG-2000 into the liposomal systems to allow for increased circulation times *in vivo* [51–54]. The efficacies of the various liposomal nanoDDSs were evaluated by quantifying their cytotoxic effects against cell lines with varying levels of CD44/CSPG expression (Scheme 1) and in a B16F10 mouse melanoma model system.

## 2. Materials and Methods

### 2.1. Chemicals

 All phospholipids (Cat# 850365, 840465, and 880120) and cholesterol (Cat# 700000) were purchased from Avanti Polar Lipids. All chemicals and solvents used in the syntheses of the triple-helical peptide (THP) PA and vesicles, such as methanol (Cat# 42395), chloroform (Cat# 650498), *tert*-butyl ether (Cat# E127), *N*,*N*-dimethylformamide (Cat# D119), *N*,*N*-diisopropylethylamine (Cat# AC11522), DOX (Cat# BP2516), and palmitic acid (Cat# 129700025) [CH_3_-(CH_2_)_14_-CO_2_H, designated C_16_], were from Fisher Scientific or Sigma-Aldrich. CellTiter-Glo Luminescent Cell Viability Assay kit (Gly-Phe-AFC) (Cat# AFC033) was purchased from Promega Corporation or MP Biomedicals. The appropriately protected amino acids, O-(1H-6-chlorobenzotriazole-1-yl)-1,1,3,3-tetramethyluronium hexafluorophosphate (HCTU) (Cat# 851012) and NovaPEG rink amide resin (Cat# 855047) were all obtained from EMD Biosciences. The preparation, purification, and characterization of the *α*1(IV)1263–1277 THP [(Gly-Pro-Hyp)_4_-Gly-Val-Lys-Gly-Asp-Lys-Gly-Asn-Pro-Gly-Trp-Pro-Gly-Ala-Pro-(Gly-Pro-Hyp)_4_-NH_2_] PA possessing a C_16_ tail have been described previously [48].

### 2.2. Cell Culture Conditions

The M14#5 and M14#11 human metastatic melanoma cell lines were generously provided by Dr. Barbara Mueller. The BJ foreskin fibroblasts from a melanoma patient were obtained from the American Type Culture Collection (ATCC) (Cat# CRL-2522). Cell media (Cat# MT10-013-CV) and trypan blue (Cat# ICN1691049) were obtained from Fisher Scientific or CellGro, and all reagents required for cell culture were purchased from Invitrogen. Cells were maintained in DMEM supplemented with 10% fetal bovine serum (Cat# 10437028), 50 units/mL penicillin, and 0.05 mg/mL streptomycin (Cat# 15140163). Cells were cultured with complete medium at 37°C in a humidified atmosphere of 5% CO_2_ in air. For all experiments cells were harvested from subconfluent (<80%) cultures using a trypsin-EDTA (Cat# 15400054) solution and then resuspended in fresh medium. Preparations of cells with a >90% viability, as determined by trypan blue exclusion, were used.

### 2.3. Preparation of DOX-Loaded Liposomes

The phospholipids and cholesterol were combined in fixed ratios (Table 1) and dissolved in an organic phase mixture of methanol, methyl *tert*-butyl ether, and chloroform (1 : 2 : 2.4) by vortexing for 0.5 h at room temperature. At this stage, if PA-targeted liposomes were the desired product (Table 1), the *α*1(IV)1263–1277 PA was added to the lipid organic phase mixture. The organic phase was then removed under reduced pressure by rotary evaporation, leaving a thin lipid film at the bottom of the flask which was dried overnight *in vacuo*. The phospholipid film was then rehydrated in ammonium sulfate (125 mM), and the resulting dispersion was vortexed extensively. The dispersion was then stirred for 30 min at 60°C. The maintenance of this temperature for a sustained time was necessary as the lipid tails were mobilized and thus allowed the aqueous medium to traverse the lipid bilayers. The resulting multilamellar vesicle (MLV) suspension was then subjected to 10 freeze-thaw cycles, briefly sonicated, followed by 10 cycles of extrusion at 60°C through 100 nm double-stacked polycarbonate filters using a Lipex Extruder (Northern Lipids, Inc., Vancouver, British Columbia) at pressures typically at the lower end of the 250–700 psi range. The polycarbonate filters employed in the extrusion process were obtained from SPI Supplies (West Chester, PA). The extruded liposomes were dialyzed against a 200-fold volume of 5% glucose solution with four changes overnight. DOX was actively loaded into the liposomes by the creation of an ammonium sulfate gradient [55, 56]. The DOX was prepared by dissolving 10 mg/mL in 5% glucose. An aliquot of 250 *μ*L of this solution was then added to each 0.1 mmol scale liposome batch and then incubated at 60°C for 2 h. The unencapsulated doxorubicin was separated from the DOX-loaded liposomes by dialysis against a 500-fold volume of PBS with 4 solution changes over 24–48 h. The size of liposomes was evaluated by dynamic light scattering as described [23]. Dynamic light scattering analysis, using a Zetasizer Nano Series, Nano ZG with Gateway 842GM (Malvren Instruments), was carried out at Louisiana State University (Department of Chemistry) to determine the mean diameter of the liposomes from each batch prepared (Table 1). Liposomes were used within 24 h of preparation or stored at 4°C and used within 1 week. The liposome phospholipid content was determined by the Stewart (ammonium ferrothiocyanate) assay as described previously [57–59]. The DOX concentration was determined by the measurement of absorbance at *λ* = 480 nm following liposome solubilization in 100% ethanol. To account for quenching effects, absorbance values were then compared to a standard curve generated using known concentrations of free DOX in the presence of empty liposomes with a drug : phospholipid ratio of 100 *μ*g/*μ*mol phospholipid. The DOX encapsulation efficiency was usually greater than 90%. The presence of the *α*1(IV)1263–1277 PA and DSPE-PEG-2000 in the liposomal bilayer was examined by MALDI-TOF mass spectrometry (MS) using an *α*-cyano-4-hydroxycinnamic acid matrix. The incorporation of the *α*1(IV)1263–1277 PA into liposomes was quantified by UV-visible spectroscopy using *ε*_280_ = 5579 M^−1^ cm^−1^ for Trp. The UV absorbance value for Trp was recorded in ethanol/PBS using a NanoDrop spectrophotometer (Thermo Scientific) and the concentration of the peptide determined using the Beer-Lambert law where *A* = *εlc*.

### 2.4. Liposome Stability

 The stability of the encapsulated doxorubicin in the various liposome systems was initially determined by monitoring DOX release from the vesicles (200 *μ*L of 0.5 mg/mL vesicle solution) at 4, 25, and 37°C, over time. Briefly, a fresh batch of liposomes was prepared and loaded with DOX. The unencapsulated doxorubicin was separated from the DOX-loaded liposomes by dialysis against a 500-fold volume of PBS as described in *Preparation of DOX*-*Loaded Liposomes*. The fluorescence intensity for each vesicle sample in PBS at each temperature was measured at selected time points within a 30 d period using a Spectra Max Gemini EM Fluorescent Plate Reader (Molecular Devices) at *λ*_excitation_ = 480 nm and *λ*_emission_ = 590 nm. Complete release of DOX from the vesicles at each time point yields 100% dequenching and was obtained from control ethanol-treated liposome samples. The percentage release of DOX from the vesicles was determined from the fluorescence intensity of each sample relative to 100% dequenching, which can then be expressed in terms of percentage of DOX release.

### 2.5. Cytotoxicity Assay

The cytotoxicity of all liposomal systems used in this study, as well as free DOX, on the cells was determined using the CellTiter-Glo Luminescent Cell Viability Assay. The M14#5, M14#11, and BJ cells were plated on 96-well tissue cultured treated plates corning at a density of 5 × 10^3^ cells per well and incubated for 24 h at 37°C and 5% CO_2_. The culture medium was then replaced with 100 *μ*L of medium containing various concentrations of each liposomal system or free DOX. The cells were then exposed to the drug for 3 h; the cells were washed twice with sterile PBS following drug exposure. Fresh culture medium was then added, and the incubation was continued for 24 h. After the incubation period, 100 *μ*L CellTiter Glo reagent was added to each well. The cells were allowed to incubate for an additional 3 h at 37°C and 5% CO_2_. The cytotoxicity assays were done in triplicate and were repeated at least twice in separate experiments. 

### 2.6. Tumor Growth *In Vivo*

B16F10 murine melanoma cells were prepared at the Washington University [60]. C57BL/6 mice were obtained from the Harlan Laboratories (Indianapolis, IN). Mice were housed under pathogen-free conditions according to the guidelines of the Division of Comparative Medicine, Washington University School of Medicine. The Washington University Animal Studies Committee approved all experiments.

Tumor cells (10^5^ cells/100 *μ*L in PBS) were injected subcutaneously in the neck of C57BL/6 anesthetized mice and allowed to grow 7–14 d until tumors were ~5 × 5 mm. Eight mice per treatment group were inoculated with 10^5^ tumor cells. The number of animals tested (*n*) was calculated by power analysis (probability of type I error *α* = 0.05; probability of type II error *β* = 0.20) based on previous data. This was the minimum number of animals required to achieve statistical significance. Mice inoculated with tumor cells were divided into a control (saline treated) as well as groups treated with the various DOX-loaded liposomes at doses (5 mg/kg with an average mouse weighing ~20 g) corresponding to those used previously for DOX-loaded liposomes in melanoma mouse models [22]. Liposomes or saline was injected on days 0, 3, 5, 6, and 8, with day 0 being the first day of the regimen and all animals dosed on the same days. The experiment was terminated at 11 d after initiation of treatment regimen.

Mice were anesthetized by isoflurane (2% vaporized in O_2_). Tumor size was determined by measuring the greatest length (*L*) and the greatest width (*W*) using calipers. The tumor size was calculated using the ellipsoid volume formula: 1/2 × *L* × *W* ^2^ [61].

### 2.7. Statistics

 The *P* values for cytotoxicity and tumor growth were calculated with the Student's *t*-test, two tailed by using Graph Pad Software.

## 3. Results

### 3.1. Construction and Characterization of Nontargeted and Targeted Liposomes

 We have previously determined that liposomes composed of DSPG, DSPC, and cholesterol (molar ratio 1 : 4 : 5) form a stable liposomal delivery system [23, 62, 63]. In addition, the presence of the *α*1(IV)1263–1277 PA did not affect the overall liposome stability. However, the earlier studies utilized ~1% of the *α*1(IV)1263–1277 PA [23], whereas efficient liposome-mediated targeting usually requires 5–23% of the peptide ligand [64–67]. Thus, the present study has examined the stability and efficacy of liposomes possessing either 5 or 10%  *α*1(IV)1263–1277 PA.

The liposomes prepared herein also incorporated DSPE-PEG-2000. The presence of PEG on liposomes allows for increased circulation times *in vivo* compared to conventional liposomes, which has been attributed to the reduced interactions between the liposomal surface and cells of the reticuloendothelial system (RES) [51–53].

The phospholipid concentration of all the liposome systems was 0.5 mg/mL, as verified by the Stewart Assay [57]. The sizes of the targeted and nontargeted liposomes assembled here were characterized using dynamic light scattering. Liposomes were 84–93 nm (small unilamellar vesicles; SUVs) (Table 1), allowing for valid stability comparisons between each system. This size range was previously found to be optimal for efficacious liposomal drug delivery to tumors [68–70]. 

To confirm the incorporation of the *α*1(IV)1263–1277 PA and DSPE-PEG-2000, liposomes were treated with ethanol to liberate the *α*1(IV)1263–1277 PA and PEG from the lipid bilayer. MALDI-TOF mass spectral analysis of the resulting solution produced a peak corresponding to the mass of the *α*1(IV)1263–1277 PA ([M+H]^+^ = 3813.3 Da, theoretical [M+H]^+^ = 3813.3 Da) and a comb-like distribution of peaks corresponding to DSPE-PEG-2000, with the predominant peaks covering [M+H]^+^ = 1727.9–2122.9 Da ([M+H]^+^ = 1728.8–2123.7 Da for DSPE-PEG-2000 directly from the supplier, dissolved in ethanol). UV-visible spectroscopic analysis following dialysis indicated 96% incorporation of the PA into liposomes.

### 3.2. Stability of *α*1(IV)1263–1277 PA to Proteolysis

To determine the stability of the *α*1(IV)1263–1277 PA in serum-containing conditions, 17.5 *μ*M PA was incubated at 37°C in either (a) water, (b) OptiMEM I media containing 4% FBS, (c) OptiMEM I media containing 10% FBS, 5 *μ*g/mL insulin, 5 ng/mL epidermal growth factor, and 40 *μ*g/mL bovine pituitary extract, or (d) 10% FBS in water. The samples were monitored by RP-HPLC at 0, 24, and 72 h. No hydrolysis of the *α*1(IV)1263–1277 PA was observed under these conditions (data not shown). Thus, the triple-helical nature of this ligand renders it reasonably stable to proteolysis (as has been observed for other THPs [71]).

### 3.3. Stability Comparison of DOX-Loaded Liposomes with and without *α*1(IV)1263–1277 PA

To determine the effect that the *α*1(IV)1263–1277 PA has on liposomal stability, DOX-loaded liposomes were prepared with and without 10%  *α*1(IV)1263–1277 PA. The DOX : phospholipid ratios were 1.65 : 1 (1300 *μ*g DOX : *μ*mol phospholipid) and 1.93 : 1 (1520 *μ*g DOX : *μ*mol phospholipid) for targeted [+10%  *α*1(IV)1263–1277 PA] and nontargeted [no *α*1(IV)1263–1277 PA] liposomes, respectively. Fluorescence intensity measurements for each vesicle sample at 4, 25, or 37°C were taken at selected time points over a 30 d period.

**Figure 2:**
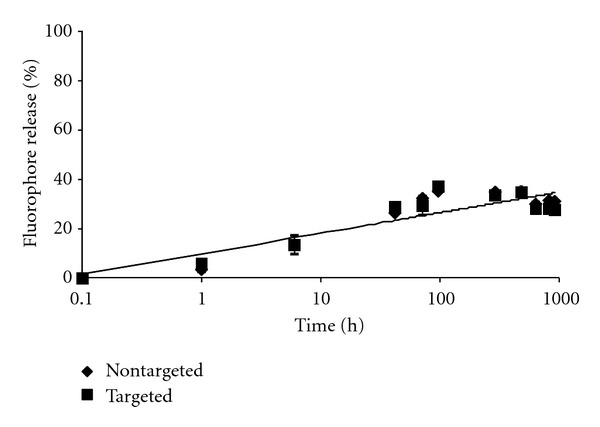
Temperature dependent stability comparisons between targeted [10%  *α*1(IV)1263–1277 PA] and nontargeted DSPG-DSPC liposomes loaded with DOX and stored at 4°C for 30 d. DOX release was determined as described in Section 2.

**Figure 3:**
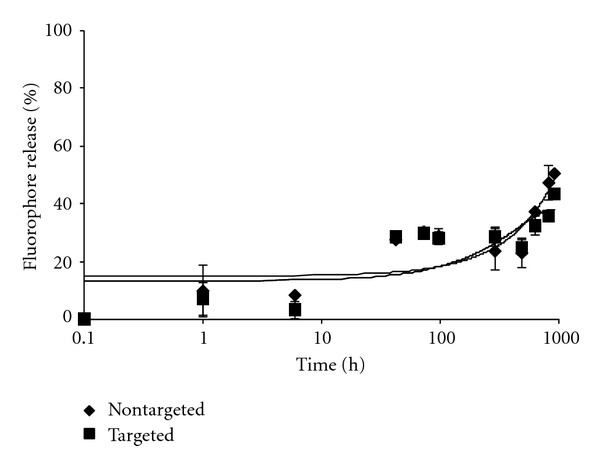
Temperature dependent stability comparisons between targeted [10%  *α*1(IV)1263–1277 PA] and nontargeted DSPG-DSPC liposomes loaded with DOX and stored at 25°C for 30 d. DOX release was determined as described in Section 2.

**Figure 4:**
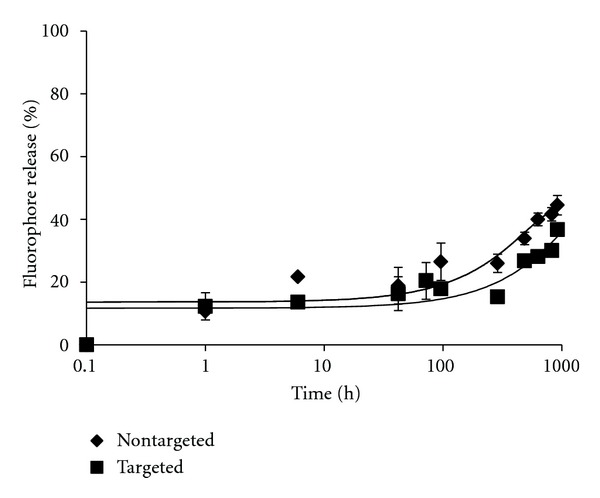
Temperature dependent stability comparisons between targeted [10%  *α*1(IV)1263–1277 PA] and nontargeted DSPG-DSPC liposomes loaded with DOX and stored at 37°C for 30 d. DOX release was determined as described in Section 2.

The targeted and nontargeted liposomes exhibited similar stability profiles over 918 h (38 d), with approximately 30–35% DOX release at 4°C (Figure 2) and 40–49% DOX release at 25 and 37°C (Figures 3 and 4). Within the first 6 h following preparation, the liposomes again demonstrated similar and minimal DOX release. Only ≤15% release was observed for both targeted and nontargeted liposomes when incubated at 4 or 25°C (Figures 2–3), and targeted liposomes were more stable than nontargeted liposomes after 6 h at 37°C (Figure 4). Data presented here are for the targeted liposomes possessing 10% PA, but similar results were observed for liposomes incorporating 5% PA (data not shown). Thus, the presence of the *α*1(IV)1263–1277 PA did not serve to destabilize the liposomes used in this study.

### 3.4. Cytotoxicity of DOX-Loaded Liposomes for Cells Varying in CD44/CSPG Content

 Cytotoxicity experiments were performed on metastatic melanoma M14#5 and M14#11 and fibroblast BJ cell lines. BJ fibroblasts have ~60% of the CD44 content of M14#5 melanoma cells, while M14#11 melanoma cells have ~75% of the CD44 content [23]. The variation in CD44/CSPG content allowed for the examination of selectivity of liposome encapsulated DOX, free DOX, and empty liposomes (Scheme 1). Empty liposomes were included due to possible unpredictable cellular responses to specific lipids within a liposome [72]. Cytotoxicity results for targeted liposomes containing 5% PA were found to be inconsistent (data not shown), so only results with 10% PA are described below.

**Figure 5:**
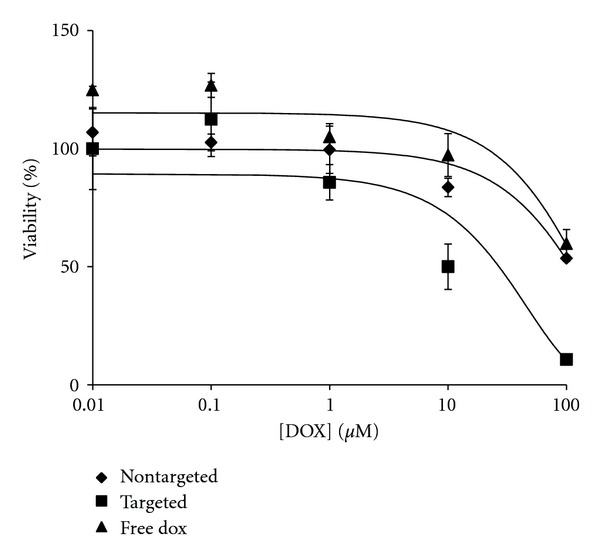
Cytotoxicity data of M14#5 cells incubated for 3 h with targeted [10%  *α*1(IV)1263–1277 PA] and nontargeted DSPG-DSPC liposomes loaded with DOX and free DOX. The difference between targeted and nontargeted liposomes loaded with DOX is statistically significant as **P = 0.00305 at 10 *μ*M and ***P = 0.00034 at 100 *μ*M.

**Figure 6:**
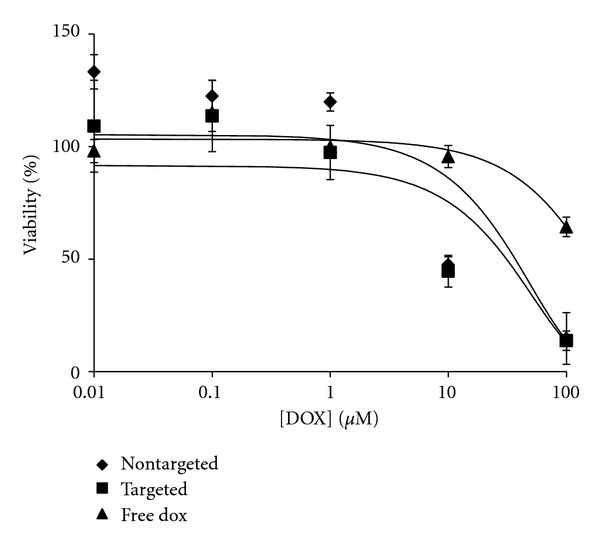
Cytotoxicity data of M14#11 cells incubated for 3 h with targeted [10%  *α*1(IV)1263–1277 PA] and nontargeted DSPG-DSPC liposomes loaded with DOX and free DOX.

A dose-dependent response was observed for M14#5 cytotoxicity by DOX encapsulated targeted liposomes (Figure 5), with an IC_50_ value of 9.8 *μ*M. Nontargeted liposomes were considerably less toxic for M14#5 cells (Figure 5) to where an IC_50_ value of 117.6 *μ*M was observed. In contrast, there was little difference in cytotoxic effects between targeted and nontargeted liposomes for M14#11 (Figure 6). More precisely, the M14#11 cell IC_50_ values for targeted and nontargeted liposomes were 9.3 and 9.9 *μ*M, respectively. Thus, the greatest difference between targeting and non-targeting was observed with the cells possessing the highest CD44 content. However, the potency of targeted liposomes with the M14#5 and M14#11 cells were relatively similar (IC_50_ values of 9.8 and 9.3 *μ*M, resp.), despite their difference in CD44 content. This may be due to cell toxicity requiring a relatively low level of DOX delivery, so, even with M14#11 cells having ~75% of the CD44 content of M14#5 cells, the amount of DOX delivered was sufficiently toxic for both cell types. The greater efficacy of nontargeted liposomes for M14#11 cells (compared with M14#5 cells) could be due to liposomal interactions with other surface molecules that are more abundant in M14#11 cells. For example, M14#5 cells express CD44 but not melanoma-associated proteoglycan/melanoma chondroitin sulfate proteoglycan (MPG/MCSP/NG2), while M14#11 cells express both [41]. Nontargeted liposomes may associate with MPG/MCSP/NG2 and thus prove more cytotoxic to M14#11 cells compared with M14#5 cells.

**Figure 7:**
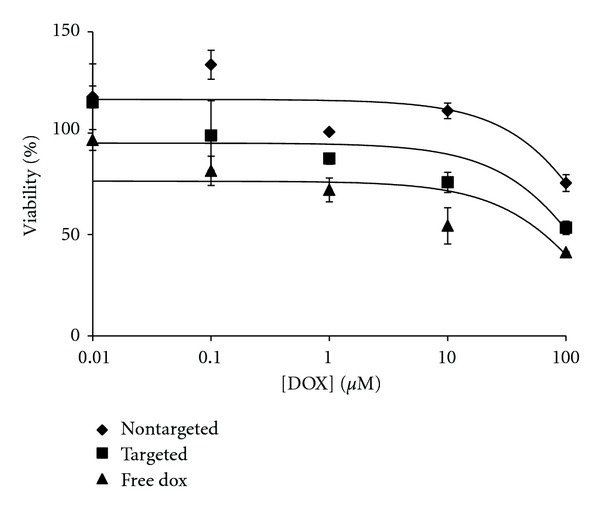
Cytotoxicity data of BJ cells incubated for 3 h with targeted [10%  *α*1(IV)1263–1277 PA] and nontargeted DSPG-DSPC liposomes loaded with DOX and free DOX.

To further evaluate the role of CD44 content in targeted delivery, the BJ fibroblast cell line was treated with free DOX and targeted and nontargeted liposomes (Figure 7). BJ fibroblasts showed a similar susceptibility to the effects of free DOX compared with the M14#5 cells (i.e., approximately 50–60% viable at [DOX] = 100 *μ*M) (Figures 5 and 7). Comparing cytotoxicities based on targeted liposomal delivery of DOX, M14#5 cells were almost completely killed at a DOX concentration of 100 *μ*M (Figure 5), while BJ cells were 60% viable (Figure 7). Thus, a positive correlation was observed between the CD44/CSPG content of M14#5 and BJ cells and the cytotoxic effects of targeted liposomes.

M14#11 melanoma cells were more susceptible to DOX than BJ fibroblasts (Figures 6 and 7). While the levels of CD44 are not the same for M14#11 cells and fibroblasts (see above), enhanced cytotoxicity made also have been influenced by different metabolic profiles of the cell types. While one presumes that the *mechanism* of DOX delivery and toxicity is same for all cell types, the metabolic rates and pathways in melanoma are different from normal cells [73], which could affect the efficiency of DOX action.

At low DOX concentrations, slight increases in cell adhesion were sometimes observed. The luminescence assay used to measure cell adhesion relies upon luciferase conversion of luciferin to oxyluciferin [74]. The luciferase activity is ATP and Mg^2+^ dependent, and thus ATP released from lysed cells directly regulates luciferase. It is possible that low concentrations of DOX could enhance luciferase activity, and thus the increase in cell adhesion is an assay artifact. If this were the case, however, one would expect the same increase in cell adhesion for all three cell types at low free DOX concentrations. This does not occur (Figures 5–7). Free DOX is only activating for M14#5 cells, while M14#11 cells and fibroblasts are activated by nontargeted liposomes. Due to the lack of a consistent trend, we believe that this slight activation is not an assay artifact. The slight activation by low levels of DOX is intriguing, but beyond the scope of the present study to further explore.

There was no significant cytotoxicity observed among the three cell lines upon incubation with empty liposomes (data not shown). Since empty liposomes were not cytotoxic, any cytotoxic effects observed here must be due solely to the cellular delivery of DOX by the respective liposomal systems.

### 3.5. Cytotoxicity of DOX-Loaded Liposomes to B16F10 Mouse Melanoma Model

**Figure 8:**
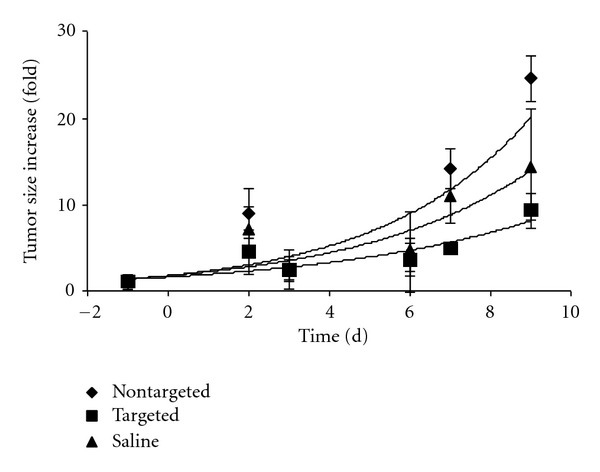
Effects of targeted [10%  *α*1(IV)1263–1277 PA] and nontargeted DSPG-DSPC liposomes loaded with DOX and saline on tumor size in the B16F10 mouse melanoma model. Liposomes or saline was injected on days 0, 3, 5, 6, and 8. On day 7 **P = 0.003 (between targeted and non-targeted) and *P = 0.0184 (between targeted and saline control); on day 9 **P = 0.0022 (between targeted and non-targeted) and *P = 0.0456 (one tail, between targeted and saline control).

 The CD44-targeted DOX-loaded PEG liposomes and nontargeted DOX loaded PEG liposomes were tested in a B16F10 mouse melanoma model. Although the B16F10 cell line is of murine origin, it highly expresses CD44 [75] and serves as a good *in vivo* model of aggressive human melanoma. Tumor size measurement was utilized to quantify the efficacy of targeted drug delivery. Mice were treated on days 0, 3, 5, 6, and 8 with 5 mg/kg DOX-loaded liposomes. Treatment with nontargeted liposomes showed no significant decrease in tumor size compared with saline control (Figure 8). However, mice treated with the targeted DOX-loaded liposomes showed substantially decreased tumor size compared with nontargeted liposomes and the saline control (Figure 8).

## 4. Discussion

We have previously constructed triple-helical *α*1(IV)1263–1277 PAs, which have been shown to be specific for CD44/CSPG [41, 47–49]. In order to develop a targeted nanoDDS specific for metastatic melanoma, *α*1(IV)1263–1277 PA has been incorporated the into liposomes [23, 62]. The results of our prior study indicated that liposomes composed of DSPG, DSPC, and cholesterol (molar ratio 1 : 4 : 5) were the most suitable for *in vitro* and *in vivo* applications [23, 63]. These liposomes proved to be the most stable of the systems tested, and the presence of the *α*1(IV)1263–1277 PA did not affect the liposomal stability. Results obtained through a series of competitive displacement experiments verified CD44/*α*1(IV)1263–1277 PA liposome recognition [23, 62]. More specifically, *α*1(IV)1263–1277 PA liposomal rhodamine delivery correlated with cellular CD44 content and was inhibited in a dose-dependent fashion by exogenous *α*1(IV)1263–1277 PA [23]. Fluorescence microscopy revealed localization of *α*1(IV)1263–1277 PA liposomes to CD44-positive cells [62].

In the present study, we further modified DSPG/DSPC liposomes with the addition of PEG. Such modifications have previously been shown to increase liposome circulation times *in vivo* [53, 76–82]. We used 5 mol % of PEG-2000 in our liposomes (Table 1), the same amount of PEG used in the clinically approved drug Doxil (DOX encapsulated PEG-stabilized liposomes) [83]. The size of the PEG chain chosen took into account the size of the PEG used in Doxil (PEG-2000) [83], as well as the impact PEGs of various sizes could have on our system specifically. Previous studies suggested that increased circulation times can be achieved with increasing PEG chain lengths up to PEG-5000 [77, 84, 85]. However, we chose not to utilize PEG larger than 2000 Da for three reasons. First, it has been shown that rigid liposomes composed of DSPC (as is the case here) exhibit a drop off in circulation times when PEG greater than 2000 Da is incorporated due to chain entanglement and lipid phase separation resulting in increased opsonization [85–88]. Second, previous work using membranes containing a mixture of the *α*1(IV)1263–1277 PA and PEGs of various sizes resulted in binding of M14#5 human melanoma cells when PEG-120, PEG-750, or PEG-2000 were used, but not with PEG-5000 [89]. Neutron reflectivity data revealed head group lengths of 8.8, 9.0, and 16.8 nm for *α*1(IV)1263–1277 PA, DSPE-PEG-2000, and DSPE-PEG-5000, respectively [89]. The lack of binding observed with PEG-5000 was thus attributed to the complete masking of the *α*1(IV)1263–1277 PA by the PEG, thereby minimizing ligand accessibility. Third, the presence or absence of 5% PEG-2000 in *α*1(IV)1263–1277 PA/DMPC (1 : 19) liposomes had little effect on the delivery of Texas Red to CD44-positive fibroblasts [62].

In the present study, cells were directly exposed to each liposomal system and free DOX and incubated at 37°C. In this environment, free DOX can be taken up by cells more rapidly than liposome encapsulated DOX. However, free DOX was not as efficacious as CD44 targeted liposome encapsulated DOX towards M14#5 melanoma cells (Figure 5). Thus, the targeting strategy promoted more efficient DOX delivery *in vitro*. Further supporting this conclusion was the observed correlation between the cytotoxic effect of DOX-loaded targeted liposomes and CD44/CSPG content for M14#5 and BJ cell lines.

Eliaz and Szoka Jr. developed CD44-targeted liposomes using HA fragments (see Section 1) [20]. Following a 3 h treatment of B16F10 mouse melanoma cells with DOX encapsulated HA liposomes, IC_50_ values of 0.78–3.62 *μ*M were observed [20]. The IC_50_ value for our CD44-targeted liposome is slightly higher (approximately 9-10 *μ*M), but we have examined activity against a highly aggressive human melanoma cell line. In addition, as discussed earlier, using HA as a targeting moiety suffers from reduced selectivity as (a) the cell surface receptor RHAMM binds to HA just as avidly as CD44 [28, 29] and (b) HA binding to CD44 is not sensitive to distinct glycosylation patterns of this receptor, while *α*1(IV)1263–1277 PA binding is [41]. Eliaz and Szoka Jr. reported an IC_50_ value for nontargeted PEG liposomes of >172.4 *μ*M, similar to what we observed for nontargeted PEG liposomes with M14#5 melanoma cells (117.6 *μ*M; Figure 5).

Potential DOX delivery *in vivo*, however, is quite different than *in vitro* when one considers circulation times. Unlike DOX encapsulated within PEGylated liposomes, free DOX is rapidly cleared from circulation, and therefore exposure to tumor cells is limited. In fact, it has previously been reported that free DOX is cleared 450-times faster than DOX encapsulated within PEGylated liposomes [90, 91]. Furthermore, extravasated PEGylated liposomes experience enhanced retention within the tumor site, which has been attributed to a lack of functional lymphatic drainage in tumors [51, 92]. In the B16F10 mouse melanoma model, DOX incorporated within nontargeted liposomes showed little effect in reducing tumor size, while targeted liposomes significantly reduced tumor size (Figure 8). The improved activity was due to the selective uptake of targeted liposomes by CD44-expressing cells rather than DOX released from disintegrated liposomes, as the targeted liposomes were more effective than the nontargeted liposomes (Figure 8), while both liposome types were of similar stability (Figures 2–4). The liposomal formulation utilized here has been noted previously as being highly stable compared with other liposomal compositions [63].

Several prior studies have examined the efficacy of DOX encapsulated, targeted liposomes on mouse tumor models [22, 24, 93]. Most relevant to the present study, Peer and Margalit compared DOX encapsulated HA liposomes, DOX encapsulated liposomes, and saline [22]. Mice were injected with C-26 colorectal tumor cells and treated at 4, 12, and 19 days with 10 mg/kg DOX. At day 31, tumor sizes were ~100, ~400, and ~1250 mm^3^ for the HA liposome, liposome, and saline treatments. Thus, CD44 targeting via HA appeared to be effective. The relative reduction in tumor size by the HA liposomes compared with saline (~12.5-fold) was greater than seen here (~2-fold; Figure 8), but the DOX dose in the prior study was twice that of our treatments (10 mg/kg versus 5 mg/kg) and the tumor type was different (colorectal versus melanoma). It should be noted that the B16F10 tumor is highly aggressive, with a doubling time of less than 24 h. Interestingly, the difference in activity for the HA liposomes and liposomes (~4-fold) [22] was comparable to that observed here for the CD44-targeted and nontargeted liposomes (~3-fold; Figure 8).

Goren et al. utilized folate-targeted liposomes for treatment following injection of M109R-HiFR lung tumor cells into mice [93]. Tumor cells were pretreated with liposomes ([DOX] = 10 *μ*M) and injected. The tumor weights after 35 days were 381 mg for untreated mice, 397 mg for mice treated with PEG liposomes (Doxil), and 57 mg for mice treated with folate-targeted liposomes. The relative reduction in tumor size by the folate-targeted liposomes compared with untreated mice (~6.7-fold) was also greater than that observed here. However, a significant difference between our study and that of Goren et al. is the injection of the tumor cells after pretreatment with liposomes in the latter case. One would anticipate that the liposomes would have a greater effect on tumor growth if they interacted with the tumor cells prior to the initiation of the tumor *in vivo*.

An apparent anomalous result from our study was the increased tumor size following nontargeted liposome treatment compared with saline control (Figure 8). Prior studies have typically reported the opposite result. For example, Charrois and Allen compared DOX encapsulated Stealth (PEG) liposomes with saline control for treatment of 4T1 mouse mammary carcinoma [70]. Saline or 6 mg/kg DOX encapsulated liposome was administered at day 4. At day 23, the tumor sizes were ~500 mm^3^ for the saline treated mice and ~80 mm^3^ for the liposome treated mice. In similar fashion, Han et al. compared DOX encapsulated PEG liposomes, DOX encapsulated comb-like polymer-incorporated liposomes, and PBS control for treatment of B16F10 inoculated mice [94]. Mice were treated at day 6 with 6 mg/kg DOX. At day 13, the tumor sizes were 300 mm^3^ for PBS control and 50 mm^3^ for the PEG liposomes and comb-like polymer liposomes. It is worth noting that, in our study, the differences between nontargeted liposomes and saline control were small at day 7 (Figure 8), which is similar to the result of Goren et al. reported above [93]. Also, the result at day 9 for the saline control is skewed lower due to one mouse treatment in which the tumor size decreased compared to day 7.

The nanoDDS described in the present study possesses several features to enhance drug selectivity and availability. The targeting capabilities rely upon a ligand that is uniquely selective for the CSPG-modified form of CD44 [41]. Although modeled after a collagen-derived sequence, *α*1(IV)1263–1277 PA is not recognized by the collagen-binding integrins found in melanoma (*α*1*β*1, *α*2*β*1, and *α*3*β*1). Thus, promiscuous receptor binding is avoided, unlike the use of HA for targeting CD44. The triple-helical nature of the ligand renders it reasonably stable to proteolysis, especially compared to other targeting molecules. The nanoDDS can also incorporate PEG to improve circulation time while minimally compromising cytotoxic activity. In principle, multitargeting can be achieved by straightforward incorporation of additional PA ligands. Multitargeting may be especially advantageous for imaging and/or therapy of cancer stem cells, where targeting of only one cell surface biomarker may not encompass the full population [16]. Thus, PA targeted liposomes may represent the “next generation” of liposomal nanoDDSs [3, 51] that have potential to enhance selectivity and targeting of chemotherapeutic treatments against metastatic melanoma in the human body. Information from these initial *in vivo* studies can guide us to improve the design of the targeted delivery vehicles.

## Figures and Tables

**Table 1:** Liposomal systems utilized for stability and cytotoxicity evaluations.

Liposome formulation	Molar ratio	Liposome diameter (nm)
Distearoyl phosphatidylglycerol (DSPG)	1	
Distearoyl phosphatidylcholine (DSPC)	4	
Cholesterol	5	84 ± 10
Distearoyl phosphatidylethanolamine		
poly(ethyleneglycol) 2000 (DSPE-PEG-2000)	0.5	

Distearoyl phosphatidylglycerol (DSPG)	1	
Distearoyl phosphatidylcholine (DSPC)	4	
Cholesterol	5	93 ± 10
*α*1(IV)1263-1277 peptide-amphiphile (PA)	0.5–1	
Distearoyl phosphatidylethanolamine		
poly(ethyleneglycol) 2000 (DSPE-PEG-2000)	0.5	
